# A new species of micro-mangrove crab of the genus *Haberma* Ng & Schubart, 2002 (Crustacea, Brachyura, Sesarmidae) from Hong Kong

**DOI:** 10.3897/zookeys.662.11908

**Published:** 2017-03-21

**Authors:** Stefano Cannicci, Peter L. K. Ng

**Affiliations:** 1 The Swire Institute of Marine Science and the School of Biological Sciences, The University of Hong Kong, Pokfulam Road, Hong Kong, Hong Kong S.A.R.; 2 Lee Kong Chian Natural History Museum, Faculty of Science, National University of Singapore, 2 Conservatory Drive, Singapore 117377, Republic of Singapore

**Keywords:** Crab, Hong Kong, new species, Sesarmidae, subtropical mangroves, taxonomy

## Abstract

The sesarmid genus *Haberma* Ng & Schubart, 2002, currently contains two species of small mangrove crabs with the first two pairs of the male ambulatory legs possessing characteristic subchelate dactyli and propodi. A new species, *H. tingkok*, is here described from Hong Kong. It can be separated from *H. nanum* Ng & Schubart, 2002 (from Singapore), and *H. kamora* Rahayu & Ng, 2005 (from Indonesian Papua) by its carapace shape, proportions of the ambulatory legs, and structures of the male pleon and male first gonopod.

## Introduction

The genus *Haberma* was established by Ng and Schubart (2002) for a small species of mangrove sesarmid crab from Singapore, *H. nanum*; and was characterised by an entire lateral carapace margin, the absence of stridulatory ridges and tubercles on the male chela and dactylus, and the possession of subchelate male first and second ambulatory legs. A second species, *H. kamora* Rahayu & Ng, 2005, was subsequently described from Papua in Indonesia. In this paper, a third species from mangroves in Hong Kong is described.

The sesarmid fauna of Hong Kong is not well studied. Stimpson (1858, 1907) reported four species of Sesarmidae Dana, 1851, s. str., and Shen (1940) recognised only seven species from the territories. Soh (1978) was the first to do a major study, recording 15 species of which four were new records and three were new species. Of the new species, Naruse and Ng (2008) synonymised *Holometopus serenei* Soh, 1978, under *Chiromantes haematocheir* (De Haan, 1833). One recent taxonomic change is recognising what had been called “*Parasesarma plicatum* (Latreille, 1803)” as *Parasesarma affine* (De Haan, 1837) instead (Rahayu and Ng 2010). *Perisesarma maipoensis* (Soh, 1978), previously regarded as a Hong Kong endemic, is now also known from Vietnam (Ng et al. 2010). Rahayu and Ng (2005: 24) recorded *Parasesarma tripectinis* (Shen, 1940) from Hong Kong, and noted it was a senior synonym of *Parasesarma acis* Davie, 1993 (see also Ng et al. 2008). It can be expected that with more intensive surveys, additional species of Sesarmidae will be discovered.

The terminology used follows that in Ng et al. (2008) and Davie et al. (2015). Measurements, in millimetres, provided are of the maximum carapace width and length, respectively. The following abbreviations are used: **G1** = male first gonopod; **G2** = male second gonopod; **P2**–**P5** = pereopods 2–5 (ambulatory legs 1–4). Material examined is deposited in the Swire Institute of Marine Science of the University of Hong Kong (**SWIMS**); the Zoological Reference Collection of the Lee Kong Chian Natural History Museum, National University of Singapore (**ZRC**); and the Zoological Museum of Florence University (**MZUF**).

## Systematics

### Family Sesarmidae Dana, 1851

#### Genus *Haberma* Ng & Schubart, 2002

##### Haberma tingkok sp. n.

Taxon classificationAnimaliaDecapodaSesarmidae

http://zoobank.org/680B85B7-8764-4332-9882-EB1CA05635AF

**Figure 1.**
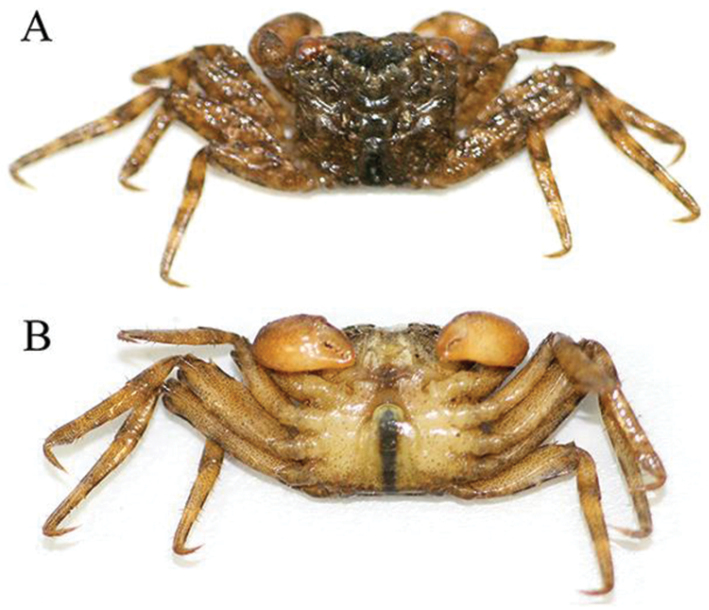
*Haberma tingkok* sp. n., colour in life, holotype ♂ (8.5 × 8.2 mm) (ZRC 2016.620). **A** dorsal view **B** ventral view.

**Figure 2.**
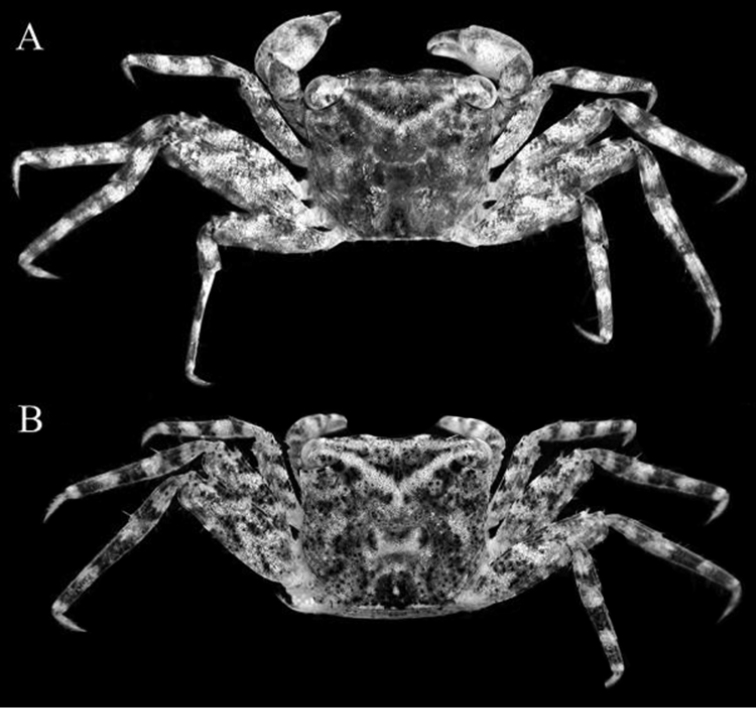
*Haberma tingkok* sp. n., overall dorsal view. **A** holotype ♂ (8.5 × 8.2 mm) (ZRC 2016.620) **B** paratype ♀ (8.6 × 8.3 mm) (ZRC 2016.621).

**Figure 3.**
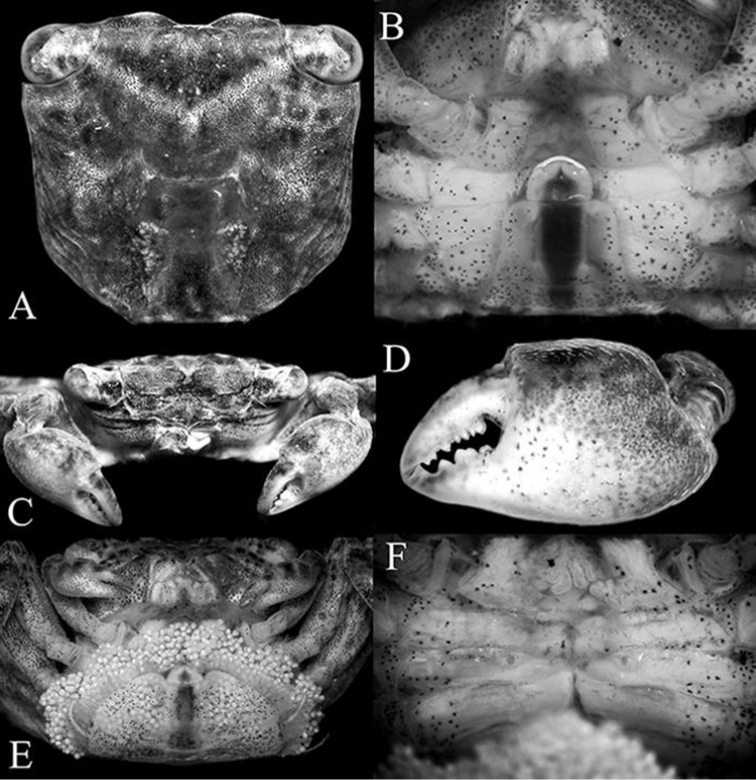
*Haberma tingkok* sp. n. **A–D** holotype ♂ (8.5 × 8.2 mm) (ZRC 2016.620) **E, F** paratype ♀ (8.6 × 8.3 mm) (ZRC 2016.621). **A** dorsal view of carapace **B** male anterior thoracic sternum and pleon **C** frontal view of cephalothorax and chelipeds **D** outer view of left chela **E** female pleon **F** female sternopleonal cavity showing vulvae.

**Figure 4.**
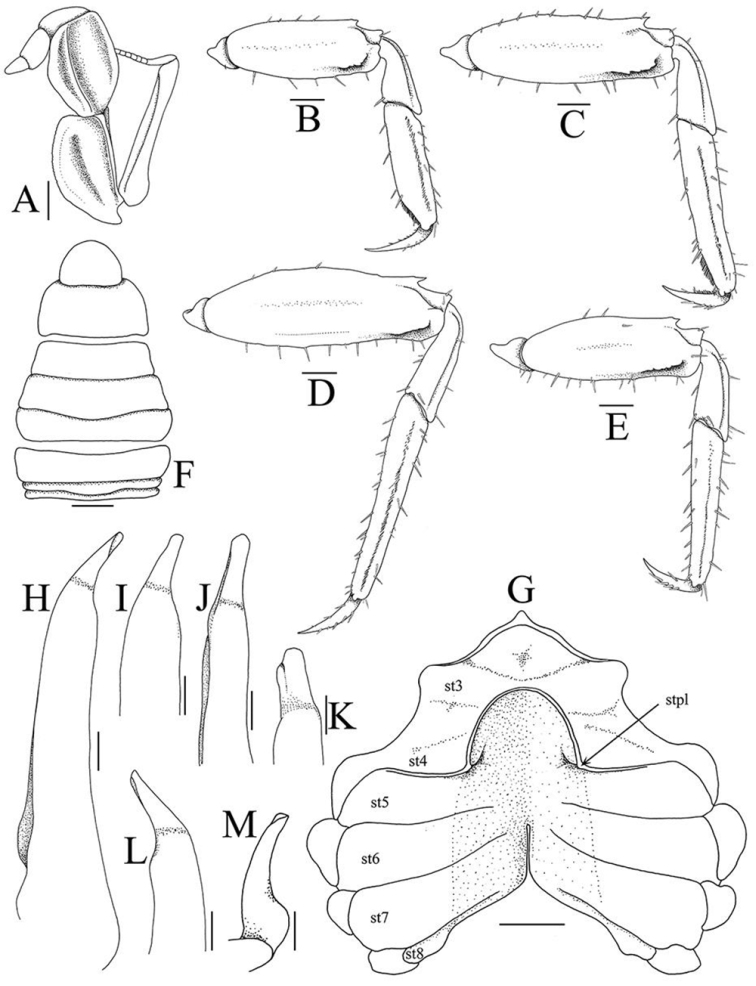
*Haberma tingkok* sp. n., holotype ♂ (8.5 × 8.2 mm) (ZRC 2016.620). **A** left third maxilliped **B–E** right P2–P5, respectively **F** pleon **G** thoracic sternum showing sternopleonal cavity **H** left G1 (dorsal view) **I** distal part of left G1 (dorsal view) **J, K** mesial view of distal part of left G1 **L** distal part of left G1 (ventral view) **M** left G2. Setae on third maxilliped, pleon and G1 denuded. Abbreviations: st3–8 = thoracic sternites 3–8, respectively; stpl: sternite 4 pleonal lock. Scale bars: **A** = 0.5 mm; **H–M** = 0.25 mm; **B–E, F, G** = 1.0 mm.

**Figure 5.**
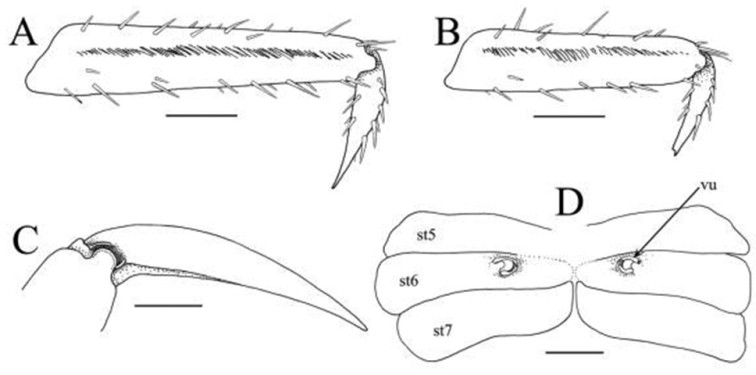
*Haberma tingkok* sp. n., paratype ♀ (8.6 × 8.3 mm) (ZRC 2016.621). **A** right P2 propodus and dactylus **B** right P3 propodus and dactylus **C** right P4 dactylus and propodus showing dactylo-propodal lock (setae not drawn) **D** thoracic sternites 5–7, showing vulvae on sternite 6. Abbreviations: st5–7 = thoracic sternites 5–7, respectively; vu: vulva. Scale bars: **A, B, D** = 1.0 mm; **C** = 0.5 mm.

Figs 1 , 2 , 3 , 4 , 5

###### Material examined.

Holotype: ♂ (8.5 × 8.2 mm) (ZRC 2016.620), Ting Kok Mangroves, Tai Po District, northeastern Hong Kong, ca. 22.28°N, 114.12°E, coll. Cannicci S, Cheung C, 4 July 2016. Paratypes: 2 ovigerous ♀♀ (8.6 × 8.3 mm, 9.2 × 9.0 mm) (ZRC 2016.621), 1 ovigerous ♀ (8.4 × 8.1 mm) (SWIMS), 1 ovigerous ♀(7.9 × 7.5 mm) (MZUF 4853), same location and date as holotype, coll. Cannicci S, Wong S; 1 ♂ (8.1 × 8.0 mm) (SWIMS), 1 young ♂ (4.6 × 4.5 mm) (ZRC 2016.622), same location as holotype, coll. Huang C, Hayhoe L, 10 August 2016.

###### Comparative material.

***Haberma nanum* Ng & Schubart, 2002**: Holotype ♂ (7.1 × 6.6 mm) (ZRC 2002.217), Mandai mangroves, Singapore, coll. Schubart CD, 29 December 1999; paratypes: 1 ovigerous ♀ (8.1 × 7.5 mm) (ZRC 2002.218), Mandai mangroves, Singapore, coll. Schubart CD, 29 December 1999; 2 ♂♂ (7.0 × 6.9 mm, 6.0 × 5.5 mm) (ZRC 2002.219), Mandai mangroves, Singapore, coll. Yeo DCJ, 16 January 2002; 4 ♂♂ (largest 6.5 × 6.5 mm, smallest 4.5 × 4.5 mm), 2 ♀♀ (5.7 × 5.8 mm, 5.1 × 4.9 mm) (ZRC 2000.1956), Mandai mangroves, Singapore, coll. Schubart CD, 15 October 1999; 1 ♂ (5.0 × 4.9 mm) (ZRC 1971.11.5.6), Johor Strait, Singapore, coll. Soh CL, 9 December 1966; 1 ♂ (6.2 × 5.8 mm) (ZRC 1971.11.5.7), Johor Strait, Singapore, coll. Soh CL, 26 December 1966; 1 dismembered ♂ (7.2 × 7.5 mm) (ZRC 1971.11.5.8), Ama Keng River, Singapore, coll. Soh CL, 28 November 1965; 1 ovigerous ♀ (8.8 × 8.3 mm) (ZRC 1968.4.22.5), Johor Straits, Singapore, coll. Soh CL, 26 December 1966; 2 ♀♀ (5.2 × 5.2 mm, 3.3 × 3.1 mm) (ZRC 1968.4.22.2-3), Sungai Melayu, Singapore, coll. Soh CL, 24 January 1966; 3 ovigerous ♀♀ (4.5 × 4.1 mm, 4.2 × 4.1 mm, 3.7 × 3.7 mm) (ZRC 1968.4.22.6-8), Johor Straits, Singapore, coll. Soh CL, 26 December 1966; 1 ovigerous ♀ (7.2 × 6.9 mm) (ZRC 1968.4.22.9), Mak Wai River, Singapore, coll. Soh CL, 28 December 1966. ***Haberma kamora* Rahayu & Ng, 2005**: Paratypes: 1 ♂ (ZRC 2000.1884), Kamora, coll. Volosin J, 4 April 2000; 4 ♂♂ (8.4 × 7.4 mm, 7.5 × 6.8 mm, 7.2 × 6.7 mm, 6.8 × 5.8 mm), 3 ♀♀ (6.4 × 5.8 mm, 6.1 × 5.6 mm, 7.9 × 7.1 mm) (ZRC 2002.0591), Kamora, near river bank, coll. Ermayanti I, 9 October 2001.

###### Diagnosis.

Carapace almost quadrate, width ca. 1.03–1.04 times length (Figs 1A, 2, 3A); margin of each frontal lobe with broadly convex margin (Figs 2, 3A); supraorbital margin relatively long, gradually sloping laterally (Fig. 3A); external orbital tooth directed anteriorly, lateral carapace margin gently sinuous, subparallel (Fig. 3A); tip of cornea reaching tip of external orbital tooth (Fig. 3A, C); dorsal surface of dactylus of chela with several small, low, irregularly arranged subtransverse striae (Fig. 3D); ambulatory legs (P2–P5) very long, slender, propodi of P2 and P3 elongate (Figs 4D, E); male pleon triangular, telson semicircular (Figs 3B, 4F); G1 relatively slender, gently curving outwards; apical process bent, with truncate tip (Fig. 4H–L).

###### Description.

Carapace almost quadrate, ca. 1.03–1.04 times broader than long; regions prominently defined, grooves separating them distinct; lateral part of branchial surface with distinct oblique striae, lined with short stiff setae; dorsal surface with scattered tufts of short setae, notably on anterior regions, lateral margins with dense short setae (Figs 1A, 2, 3A). Postfrontal regions distinct, distinctly separated into 4 lobes by deep grooves, median lobes larger than lateral ones (Fig. 3A). Front ca. 0.5 times maximum carapace width, prominently deflexed, margin distinctly bilobed from dorsal view, each lobe with broadly convex margin, separated by broad median concavity (Figs 2, 3A). Supraorbital margin relatively long, gradually sloping laterally, gently convex, entire (Fig. 3A). External orbital tooth triangular, directed anteriorly, completely fused with lateral carapace margin without trace of tooth or indentation; lateral carapace margin gently sinuous, subparallel; posterior carapace margin almost straight (Fig. 3A). Eyes and orbits large, eye longer than orbit, tip of cornea reaching tip of external orbital tooth (Fig. 3A, C). Basal articles of antenna adjacent to antennule, not separated by septum; basal antennal article large, flagellum short, entering orbit; basal antennular article bulbous, antennule folding obliquely (Fig. 3C). Posterior margin of epistome with prominent median triangular projection, lateral margins conspicuously sinuous (Fig. 3C).

Ischium of third maxilliped with shallow, oblique median sulcus; merus shorter than ischium, with distinct oblique median ridge; exopod slender, tip reaching to more than half length of outer margin of merus, flagellum long (Fig. 4A).

Chelipeds subequal; male chelipeds relatively stout (Figs 2A, 3D); female chelipeds distinctly more slender (Fig. 2B). Basis-ischium fused, suture visible, inner margin gently serrated; posterior border of merus serrated, with low subdistal tooth; outer anterior border gently serrated with distinct proximal spine; inner anterior border gently serrated with prominent subdistal tooth; carpus subovate, longer than broad, margins subcristate, with tuft of setae on inner margin (Figs 2A, 3B). Outer and inner surfaces of palm smooth to rugose, without setae (Fig. 3D). Dorsal surface of palm with several short uneven ridges lined with very small rounded granules, not prominently raised or pectinated (Fig. 3D). Male propodal finger short, dactylus curved, smooth on outer surfaces; dorsal surface of dactylus with several small, low, irregularly arranged subtransverse striae; cutting edge of fingers with small and large teeth, tips chitinous, subspatuliform; fingers forming small gape when closed (Fig. 3D). Female fingers less distinctly curved, without striae on dorsal margin of dactylus (Fig. 3E).

Ambulatory legs (P2–P5) very long, slender, third pair longest (Fig. 2). Outer surface of merus, carpus and propodus gently rugose to smooth (Figs 2, 4B–E). Meri ca. three times as long as wide; dorsal margin gently serrated, with sharp subdistal spine; ventral margin more distinctly serrated; dorsal and ventral margins with regularly arranged long stiff setae; outer surface with short, uneven ridge on subdistal part (Fig. 4B–E). Carpus with two subparallel ridges on outer surface, one marginal, one submarginal (Fig. 4B–E). Male propodus of P2, P3 with ventro-distal margin sharply tapering at distal quarter; dorsal and ventral margins with prominent long stiff setae, outer surface with longitudinal median row of short dense setae; disto-ventral margin with dense short brush-like setae, with long, stiff setae bracketing brush; dactylus of P2, P3 styliform, gently curving, proximal half with short brush-like setae along ventral margin; dactylus folding against tapered part of propodus when flexed, brush-like setae of each appendages appressing tightly against each other, forming distinct subchelate structure (Fig. 4B, C). Female propodi and dactyli of P2, P3 not subchelate; brush-like setae on ventral margins absent (Fig. 5A, B). Propodi and dactyli of female P2, P3, and male and female P4, P5 normal, not subchelate, dorsal and ventral margins with prominent long stiff setae; propodus elongate, outer surface with median longitudinal row of setae; dactylus distinctly shorter than propodus, styliform, gently curved (Figs 4D, E, 5A, B). Dactylus of all legs of both sexes with distinct dactylo-propodal locking mechanism (Fig. 5C)

Surface of male thoracic sternites 1–3 setose, others smooth, glabrous; sternites 1–3 fused; sternites 3 and 4 separated by very low ridge lined with long setae that obscure margins (Figs 3B, 4G). Male sternopleonal cavity reaching low ridge separating sternites 3 and 4 (Figs 3B, 4G). Tufts of long, soft setae between coxae of chelipeds and first to third ambulatory legs, those between ambulatory legs denser. Male pleonal locking mechanism formed by slightly raised edge of ridge on sternite 4, at edge of sternopleonal cavity between sternites 4 and 5; no trace of locking tubercle or granule (Fig. 4G). Vulva on submedian part of sternite 6, with operculum and distinct opercular processes (Figs 3F, 5D).

**Figure 6.**
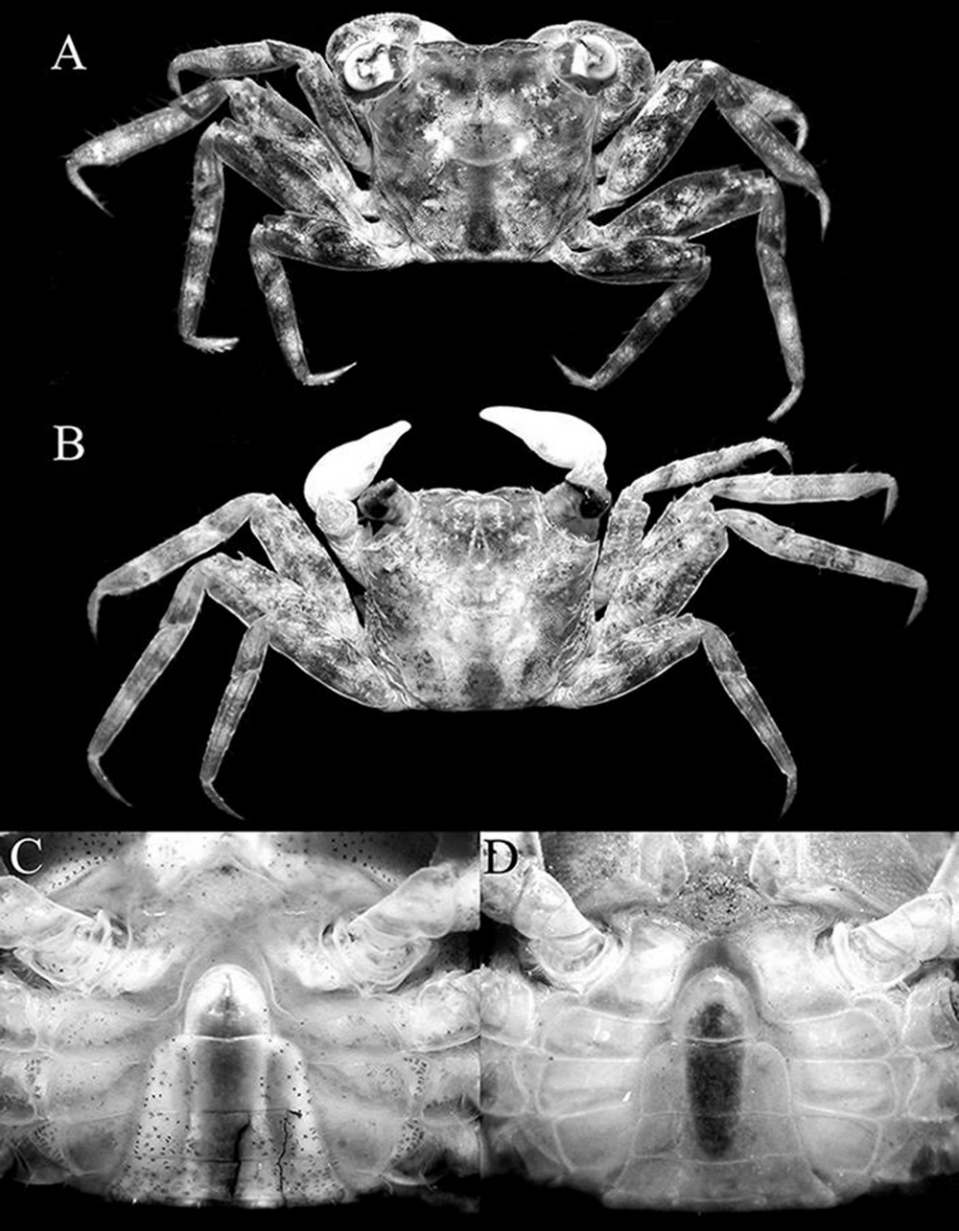
**A, C** *Haberma nanum* Ng & Schubart, 2002, holotype ♂ (7.1 × 6.6 mm) (ZRC 2002.217), Singapore **B, D** *Haberma kamora* Rahayu & Ng, 2005, paratype ♂ (7.5 × 6.8 mm) (ZRC 2002.591), Indonesian Papua. **A, B** overall dorsal view **C, D** anterior thoracic sternum and pleon.

Male pleon triangular, relatively broad (Figs 3B, 4F); telson semicircular, shorter than somite 6, lateral margin convex; somite 6 twice as long as wide, lateral margins distinctly convex; somites 4 and 5 progressively more trapezoidal, lateral margins almost straight; somite 3 broadest, lateral margin convex; somites 1 and 2 longitudinally narrow (Figs 3B, 4F). Female pleon almost round, almost completely covering thoracic sternal surface (Fig. 3E). Male thoracic sternite 8 not visible when pleon closed.

G1 relatively slender, gently curving outwards; chitinous apical process bent, relatively long, with truncate tip; subdistal setae long, simple, at base of apical process (Fig. 4H–L). G2 short with spatuliform tip (Fig. 4M).

Eggs small, subovate (Fig. 3E), ca. 0.25 mm in freshly preserved material.

###### Variation.

The adults examined do not vary substantially. The smallest specimen, a young male measuring 4.6 × 4.5 mm (ZRC 2016.622), differs from the adult males in having the lateral margins of the carapace gently concave, resembling the condition observed in *H. kamora* (Fig. 6B) but the carapace still looks relatively quadrate and the supraorbital margin is less sloping. Its subchelate P2 and P3 are less prominent, mainly because the setae on the distal margin of the propodus and that of the dactylus are less developed, being very short. In addition, its pleon is less semicircular in shape, being more triangular and closer to that observed in *H. kamora* (Fig. 6D).

###### Colour.

In life, carapace dark brown with light brown mottling; ambulatory legs mottled brown with darker bands on carpi and propodi; chelae light-orange to orange, with fingers darker coloured (Fig. 1A, B). Ventral surfaces of cephalothorax and ambulatory legs light yellow with numerous fine brown spots (Fig. 1B).

###### Etymology.

The species is named after the Ting Kok mangrove area, which has been designated a “Site of Special Scientific Interest” in Hong Kong. The name is used as a noun in apposition.

###### Remarks.

*Haberma tingkok* sp. n. can easily be separated from *H. nanum* Ng & Schubart, 2002, and *H. kamora* Rahayu & Ng, 2005, by the carapace appearing proportionately broader (Fig. 3A) rather than more distinctly quadrate (Fig. 6A, B). While their carapace width to length proportions do not differ substantially, this difference in carapace shape is due mainly to the lateral margins of *H. tingkok* being straighter and less sinuous (Fig. 3A); which in *H. nanum* and *H. kamora*, are distinctly more sinuous (Fig. 6A, B). This also affects the shape of the supraorbital margin. In *H. tingkok*, the margin is relatively longer and gently curves laterally to the external orbital tooth (Fig. 3A). In *H. nanum* and *H. kamora*, the supraorbital margin appears shorter as it curves obliquely to the margin (Fig. 6A, B). This difference in carapace shape applies for both sexes (Fig. 2). In addition, the male P4 and P5 propodi are prominently more elongated (Fig. 2A, 4D, E) than those of *H. nanum* and *H. kamora* (Fig. 6A, B). The margin of the frontal lobe of *H. tingkok* is the most convex in the genus (Fig. 3A); in *H. nanum*, the margin is almost straight (Fig. 6A) whilst in *H. kamora*, it is gently convex (Fig. 6B). The male telson of *H. tingkok* is distinctive as it is semicircular (Figs 3B, 4F); in *H. nanum* and *H. kamora*, the telsons are relatively longer and more triangular (Fig. 6C, D). The distal chitinous part of the G1 of *H. tingkok* (Fig. 4H–L) is more curved than that of *H. nanum* (cf. Ng and Schubart 2002: fig. 4B, C); and while it is more similar in form to that of *H. kamora*, the chitinous distal part of the G1 of this species is relatively longer (cf. Rahayu and Ng 2005: fig. 3H, I).

One character not described in Ng and Schubart (2002) and Rahayu and Ng (2005) is the presence of a short, uneven ridge on the outer surface of the ambulatory merus, at the subdistal part just before the carpus (Fig. 4B–E). It is present on all the legs, being more prominent in P2 and P3. This ridge is present also in *H. nanum* and *H. kamora*, but is relatively less well developed compared to *H. tingkok*.

###### Ecology.

The specimens were found climbing trees of *Kandelia obovata* Sheue, Liu & Yong, 2003, and *Aegiceras corniculatus* (L.) Bianco, 1837, in the mid intertidal area of the Ting Kok mangrove stand, in Tolo Harbour. The area is the largest mangrove stand on the eastern coast of Hong Kong and is largely dominated by *K. obovata* trees, up to 3 m tall. All specimens, including the ovigerous females, were collected at a height of approximately 1.5–1.8 m above the substrate, walking on the bark of the branches at ebbing and low tides.

## Supplementary Material

XML Treatment for Haberma tingkok
